# Serotonin and Melatonin Biosynthesis in Plants: Genome-Wide Identification of the Genes and Their Expression Reveal a Conserved Role in Stress and Development

**DOI:** 10.3390/ijms222011034

**Published:** 2021-10-13

**Authors:** Bidisha Bhowal, Annapurna Bhattacharjee, Kavita Goswami, Neeti Sanan-Mishra, Sneh L. Singla-Pareek, Charanpreet Kaur, Sudhir Sopory

**Affiliations:** 1 International Centre for Genetic Engineering and Biotechnology, Aruna Asaf Ali Marg, New Delhi 110067, India; bidisha@icgeb.res.in (B.B.); abhattacharjee2017@gmail.com (A.B.); kavita@icgeb.res.in (K.G.); neeti@icgeb.res.in (N.S.-M.); sneh@icgeb.res.in (S.L.S.-P.); 2 Department of Biochemical Engineering and Biotechnology, Indian Institute of Technology-Delhi, New Delhi 110016, India

**Keywords:** abiotic stress, Arabidopsis, melatonin, miRNA, rice, serotonin, sorghum, tomato

## Abstract

Serotonin (Ser) and melatonin (Mel) serve as master regulators of plant growth and development by influencing diverse cellular processes. The enzymes namely, tryptophan decarboxylase (*TDC*) and tryptamine 5-hydroxylase (*T5H*) catalyse the formation of Ser from tryptophan. Subsequently, serotonin N-acetyl transferase (*SNAT*) and acetyl-serotonin methyltransferase (*ASMT*) form Mel from Ser. Plant genomes harbour multiple genes for each of these four enzymes, all of which have not been identified. Therefore, to delineate information regarding these four gene families, we carried out a genome-wide analysis of the genes involved in Ser and Mel biosynthesis in Arabidopsis, tomato, rice and sorghum. Phylogenetic analysis unravelled distinct evolutionary relationships among these genes from different plants. Interestingly, no gene family except ASMTs showed monocot- or dicot-specific clustering of respective proteins. Further, we observed tissue-specific, developmental and stress/hormone-mediated variations in the expression of the four gene families. The light/dark cycle also affected their expression in agreement with our quantitative reverse transcriptase-PCR (qRT-PCR) analysis. Importantly, we found that miRNAs (miR6249a and miR-1846e) regulated the expression of Ser and Mel biosynthesis under light and stress by influencing the expression of *OsTDC5* and *OsASMT18*, respectively. Thus, this study may provide opportunities for functional characterization of suitable target genes of the Ser and Mel pathway to decipher their exact roles in plant physiology.

## 1. Introduction

Serotonin (Ser) and melatonin (Mel) are indoleamines which are synthesised in most life forms including prokaryotes such as bacteria, as well as fungi, nematodes, animals and plants; however, their presence in archaea remains to be studied [1]. They are well known neurotransmitters, which play vital roles, especially in animals, being associated with diverse physiological processes. In humans, Ser governs behavioural patterns, i.e., moods and appetite, and Mel regulates sleep, circadian rhythms, immunity enhancement and even aids in the reduction of oxidative stress in animals [2,3]. With the emergence of plant neurobiology as an exciting field of research, investigations pertaining to the precise roles of Ser and Mel in plants have received greater attention [4]. 

**Figure 1:**
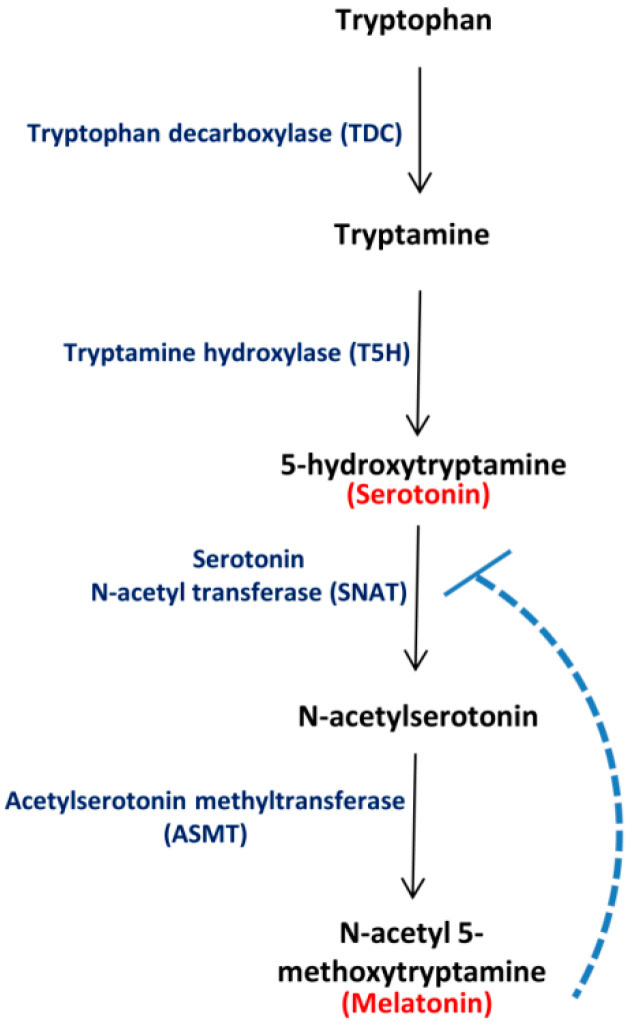
Major route for Ser and Mel biosynthesis in plants. Tryptophan decarboxylase (*TDC*) converts tryptophan to tryptamine, which is converted to 5-hydroxytryptamine (Ser) by tryptamine 5-hydroxylase (*T5H*). Serotonin N-acetyl transferase (*SNAT*) converts Ser to N-acetyl serotonin, which is further transformed to N-acetyl 5-methoxytryptamine (Mel) by acetylserotonin methyltransferase (*ASMT*). The broken line depicts feedback regulation of *SNAT* by Mel, as reported in literature.

Tryptophan, an essential aromatic amino acid, primarily acts as the common substrate for biosynthesis of various plant indoleamines, namely, Ser, Mel and auxin [5,6,7]. Cells containing high tryptophan levels, generally, accumulate greater amounts of Ser and, subsequently, Mel [8]. According to the classical pathway, tryptophan decarboxylase (*TDC*) and tryptamine 5-hydroxylase (*T5H*) enzymes are required for the production of Ser, whereas serotonin N-acetyltransferase (*SNAT*) and acetyl serotonin methyltransferase (*ASMT*) enzymes are required for Mel production from Ser in plants (Figure 1). While *TDC* is the rate-limiting enzyme of Ser biosynthesis, both *SNAT* and *ASMT* regulate Mel synthesis under specific conditions and catalyse the dedicated steps in Mel biosynthesis [3,8]. The *SNAT* enzyme is regulated by feedback inhibition in rice [9] (Figure 1). In silico studies pertaining to the genes involved in Mel biosynthesis have led to the identification of 5 genes encoding for *TDC* proteins and 14 genes encoding for *ASMT* proteins in the tomato genome [10,11]. Furthermore, the existence of *TDC* in *Citrus* is also experimentally proven [12]. 

Notably, light/dark conditions can modulate Mel levels in plants. In herbaceous peony, Mel production depends on light availability, specifically increasing at the bloom stage of its development [13]. This increase, in turn, can be correlated with enhanced gene expression patterns of the rate-limiting enzyme, *TDC* [13]. Likewise, expression levels of genes encoding for Ser and Mel biosynthetic enzymes in rice are also altered during light and dark conditions [14,15]. 

Mel regulates multiple plant physiological pathways such as growth and reproduction, senescence, chrono-regulation, root and shoot organogenesis and diverse hormonal crosstalk, as well as stress responses [16,17,18,19]. It acts as a scavenger of reactive oxygen species, reduces cellular damage in plants by repairing mitochondria and improves the shelf life of fruits and vegetables, owing to its antioxidant properties [20,21,22,23]. The multifunctional roles of Mel in plants and the recent discovery of the first plant Mel receptor has led to emerging speculations of Mel acting as a new phytohormone or master regulator [24]. Likewise, Ser also plays diverse roles in plants, with its functions in stress response being delineated in the last decade [7,25,26].

Importantly, the role of Ser and Mel in mediating stress-related signalling is also emerging [19,27,28,29,30]. Ser and Mel have been shown to regulate root growth in sunflower under salinity stress [31]. Interestingly, nitric oxide in conjunction with Mel modulates glutathione reductase activity and alters glutathione content in sunflower seedling cotyledons under salinity stress conditions [32]. Indeed, Mel along with nitric oxide acts as a salt-signalling molecule in sunflower and mediates stress responses via its antioxidant property [33]. In addition, microRNAs (miRNAs) have also been found to modulate abiotic stress responses in plants [34]. Furthermore, Mel in conjunction with a specific miRNA, miR398, has been found to mediate oxidative and heavy metal stress tolerance in plants [35].

There are several reports highlighting the roles of Ser and Mel in plant development and stress responses. However, no systematic information regarding the genetic organization of the respective genes involved in Ser and Mel biosynthesis is presently available in plants. Hence, in the current study, we have attempted to identify the genes involved in Ser and Mel biosynthesis in four model plant species, namely, Arabidopsis, rice, sorghum and tomato, followed by analysis of their evolutionary relationships. Gene expression profiles of genes encoding enzymes for Ser and Mel biosynthesis, in different tissues/organs during various stages of development, under light/dark conditions and in response to various stress factors, were also analysed. Additionally, we could even identify miRNAs that regulate the expression of genes encoding enzymes for Ser and Mel biosynthesis in selected model plants. Taken together, we provide detailed information regarding gene family members associated with Ser and Mel biosynthesis in plants. Our study can be beneficial for identifying the crucial targets needed for manipulation of Ser and Mel pathway.

## 2. Results

### 2.1. Plants Encode Multiple Isoforms of Genes Involved in Ser and Mel Biosynthesis

Genes encoding enzymes for Ser and Mel biosynthesis were identified from rice, Arabidopsis, tomato and sorghum, as described in the Materials and Methods section. As in other gene families, the genes involved in Ser and Mel biosynthesis probably encode multiple proteins. We found that 7 and 9 *TDC* genes, 2 and 1 *SNAT* genes and 19 and 28 *ASMT* genes are present in rice and sorghum, respectively (Table 1). In Arabidopsis and tomato, 2 and 5 *TDC* genes, 1 and 2 *SNAT* genes and 17 and 11 *ASMT* genes were respectively identified. On the other hand, *T5H* is present as a single gene in rice, sorghum and Arabidopsis, whereas in tomato, six isoforms of the *T5H* gene could be detected (Table 1). 

Among the *TDC*s, Arabidopsis *AtTDC2* possesses four spliced forms, whereas no spliced variants could be identified for *TDC* genes in rice, tomato and sorghum. The protein length of the identified TDCs vary from 374 amino acids (aa) to 588 aa, and their isoelectric points range between 5 and 8, with few exceptions, e.g., OsTDC3 and SbTDC9 (Supplementary Table S1). While the protein lengths of the identified T5Hs vary from 338 aa to 523 aa, their isoelectric points range between 5.9 and 8.5 (Supplementary Table S1). Among the *SNAT*s, Arabidopsis *AtSNAT1* is the only gene with three spliced variants, whereas genes from rice, tomato and sorghum do not exhibit splicing. The protein length of the identified SNATs vary from 191 aa to 258 aa, and their isoelectric points broadly range from 4.9 to 10.3 (Supplementary Table S1). Among the identified *ASMT*s, *AtASMT6* and *AtASMT8* from Arabidopsis and *SbASMT22* from sorghum have two spliced variants each. The protein lengths of the identified ASMTs is highly variable, from 126 aa to 428 aa, and the isoelectric points range between 4.6 and 7, with the exception of SlASMT6, which has an isoelectric point of 9.02 (Supplementary Table S1). Overall, the proteins involved in Ser and Mel biosynthesis in selected model plants possess highly diverse characteristics. The details of the respective proteins and their physico-chemical properties, such as molecular weight and isoelectric points and sub-cellular localization, are listed in Supplementary Table S1.

### 2.2. Proteins Specific to Mel Biosynthesis Show Greater Variations in Domain Architecture

**Figure 2:**
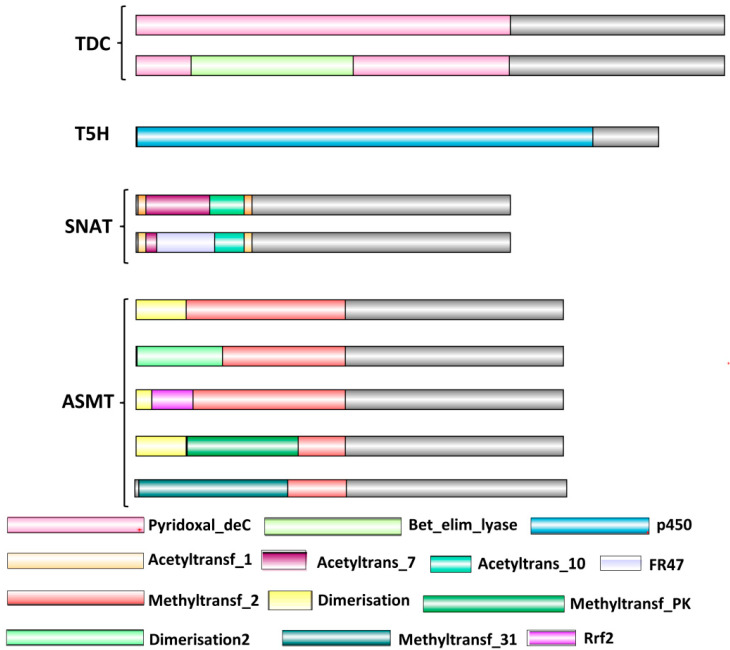
Schematic representation of domain architecture of Ser and Mel biosynthetic enzymes. Domains were analysed using the Pfam database. Lengths of domains shown are not to the scale. *TDC*, Tryptophan decarboxylase; *T5H*, Tryptamine-5-hydroxylase; *SNAT*, Serotonin-N-Acetyl transferase; *ASMT*, Acetylserotonin methyltransferase.

All enzymes related to Ser and Mel biosynthesis possess functional domains characteristic of their catalytic activities (Figure 2). TDCs possesses a typical Pyridoxal_deC domain, which is responsible for pyridoxal phosphate binding and carboxy-lyase activity. Moreover, the beta-elim-lyase domain, acting as beta-eliminating lyases with a pyridoxal-phosphate attachment site, is also found in some *TDC* proteins such as OsTDC6, SbTDC4, SbTDC5 and TDCs from Arabidopsis. In contrast, *T5H* proteins in all the analysed species were not found to harbour any additional domain besides the characteristic P450 domain, which mediates oxidoreductase activity and is also involved in heme or iron–ion binding. Further, SNATs contain an acetyltransferase domain (Acetyltransf_1/7/10) which governs the transferase activity of enzyme by transferring an acetyl group. Some *SNAT* proteins (OsSNAT1, AtSNAT1.1/1.2/1.3 and SbSNAT1) also contain the FR47 domain, which is often found at the N-terminus to the GNAT acetyltransferase domain and is a domain of unknown function (DUF481) [36]. On the other hand, ASMTs are marked by a typical methyltransferase domain (Methyltransf_2), which governs O-methytransferase activity, with most of the *ASMT* proteins also possessing dimerization domains at the N-terminus. However, AtASMT13 possesses an additional Rrf2 domain, initially detected in bacteria. It is the regulator of the high molecular weight cytochrome (hmc) operon encoding iron-sulphur-containing proteins as well as other proteins involved in electron transport [37]. Hence, the presence of typical domains in these proteins render activities characteristic of their function and thus, are crucial for mediating Ser and Mel biosynthesis, but additional domains add to the diversity and impart special features to these proteins. In this context, the enzymes involved in Mel biosynthesis exhibit greater variations in domain architecture than those facilitating Ser biosynthesis. Details of the domains present in respective proteins have been tabulated in Supplementary Table S2.

### 2.3. Enzymes of Ser and Mel Biosynthetic Pathway Exhibit Differential Phylogenetic Relationships with Varied Localization Patterns

In order to understand the phylogenetic relationships of proteins mediating Ser and Mel biosynthesis in the four plant species, their amino acid sequences were retrieved and aligned. Based on the sequence similarity obtained via the neighbour-joining (NJ) method, the *TDC* proteins were found to form three distinct clusters in the phylogenetic tree. The first cluster was composed of the *TDC* proteins from rice, tomato and sorghum, but no protein from Arabidopsis was present in this cluster. The second cluster consisted of *TDC* proteins from only rice and sorghum, whereas the third cluster comprised *TDC* proteins from all the four model plants. 

**Figure 3:**
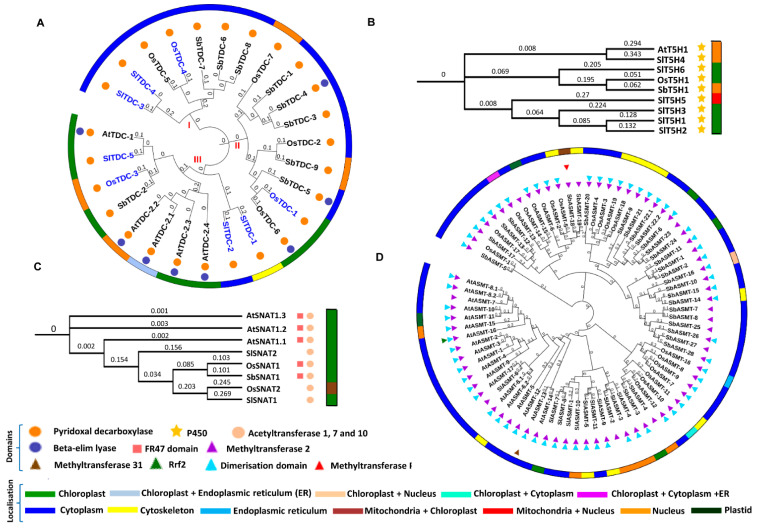
Evolutionary analysis, domain and localization details of genes encoding Ser and Mel biosynthetic enzymes. Phylogenetic tree depicting evolutionary relationship between genes encoding Ser biosynthetic enzymes, (**A**) tryptophan decarboxylase genes (*TDC)* and (**B**) tryptophan 5-hydroxylase genes (*T5H*), and Mel biosynthetic enzymes (**C**) serotonin N-acetyltransferase genes (*SNAT)* and (**D**) acetyl-serotonin methyltransferase genes (*ASMT)*, from Arabidopsis, rice, sorghum and tomato. Localization details are indicated by the coloured strip besides the phylogenetic trees (**A**–**D**). The proteins labelled in blue have been previously characterized. The branch lengths indicate evolutionary time between the two nodes.

While the characteristic pyridoxal decarboxylase domain was present in all TDCs, the beta-elim-lyase domain could only be found in some *TDC* proteins of rice and sorghum (e.g., OsTDC6, SbTDC4 and SbTDC5) but in all *TDC* proteins of Arabidopsis. Notably, Cluster I proteins and all tomato *TDC* proteins lacked the beta-elim-lyase domain (Figure 3A). The *TDC* proteins in Clusters I and II were predicted to be cytoplasmic, with the exception of some proteins of Cluster II, such as OsTDC1, OsTDC6 SbTDC9 and OsTDC7, which were predicted to be localised in the chloroplast, and nucleus, respectively. Interestingly, *TDC* proteins in Cluster III, being the most diverse, showed majorly mixed localization.

The *T5H* proteins identified in this study branched into three major subgroups in the phylogenetic tree. In the first subgroup, the Arabidopsis AtT5H1 was found to be closer to SlT5H4 from tomato. In the second subgroup, SlT5H6 clustered with OsT5H1 and SbT5H1, whereas the third subgroup was composed exclusively of the remaining four *T5H* proteins identified from tomato (Figure 3B). Three localization variants were predicted among the *T5H* proteins from the four species. While most *T5H* proteins in tomato were localized in chloroplast, AtT5H1, SlT5H4 and SbT5H1 were predicted to be localized in the nucleus. Interestingly, dual localization in mitochondria and the nucleus was predicted for SlT5H5 (Figure 3B).

As with *TDC* and *T5H* proteins, *SNAT* proteins from four plant species also grouped into three clades (Figure 3C). While AtSNAT1.3 and AtSNAT1.2 branched independently, AtSNAT1.1 and *SNAT* proteins from tomato grouped with SNATs from rice and sorghum, as depicted in the third cluster of the phylogenetic tree. All the *SNAT* proteins were predicted to be chloroplast-localized, except for OsSNAT2, which demonstrated putative chloroplastic as well as mitochondrial localization (Figure 3C).

The multiple members identified in the *ASMT* family branched into two major clusters in the phylogenetic tree, one constituting ASMTs from rice and sorghum and the other comprising Arabidopsis and tomato proteins (Figure 3D), unlike the other three types of proteins described above. Most of the *ASMT* orthologs were more closely related to members identified within each species as compared with those from other plant species. While the majority of the *ASMT* proteins were predicted to be cytoplasmic, there were also some variants. For example, *ASMT* proteins such as OsASMT5/11/18/19, SlASMT5/9, AtASMT6 and SbASMT9/14/19/21 were localized in cytoskeleton, whereas a few, such as AtASMT15, SlASMT2/3/4/10 and SbASMT4, were found to be nuclear localised (Figure 3D). Chloroplast localization was predicted in the case of SbASMT3/6/11 and SlASMT7, whereas AtASMT11 and OsASMT15 had plastidial localization (Figure 3D). Additionally, OsASMT16 was predicted to be in endoplasmic reticulum (Figure 3D). Few *ASMT* proteins showed dual localization, such as SbASMT16, which was predicted to localize in chloroplast and nucleus, whereas OsASMT10 possibly localized in chloroplast as well as cytoplasm (Figure 3D). Interestingly, OsASMT13 was predicted to be localized in cytoplasm and chloroplast, in addition to endoplasmic reticulum (Figure 3D). Overall, the analysis indicated that the proteins involved in Ser and Mel biosynthesis exhibited distinct evolutionary relationships and diverse localization patterns. In particular, chloroplast and cytoplasm were predicted as the key sites, where most of the proteins involved in Ser and Mel biosynthesis localize.

### 2.4. Expression of Genes Encoding Ser and Mel Biosynthetic Enzymes Is Highly Variable in Tissues during Development

**Figure 4:**
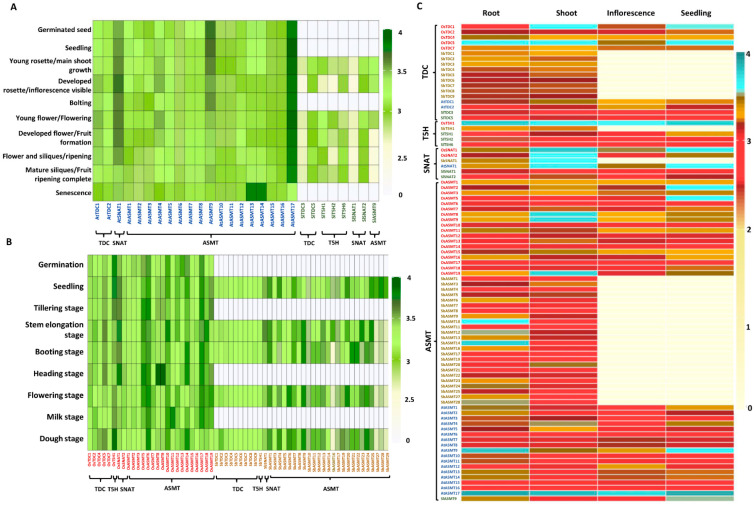
Tissue-specific and developmental regulation of genes encoding Ser and Mel biosynthetic enzymes in different plant species. Expression profile of genes encoding Ser and Mel biosynthetic enzymes in dicots (**A**), Arabidopsis and tomato, and monocots (**B**), rice and sorghum, was obtained from the publicly available Genevestigator database. Data was retrieved for different developmental stages (**A**,**B**) and different tissues (**C**), namely root, shoot, inflorescence and seedling. Colour scale on the right side of the heatmaps indicates the levels of expression. Grey/Light yellow colour indicates unavailability of data. Data pertaining to the *T5H* gene family of Arabidopsis was not available and, hence, is not shown. *TDC*, Tryptophan decarboxylase; *T5H*, Tryptamine-5-hydroxylase; *SNAT*, Serotonin-N-Acetyl transferase; *ASMT*, Acetylserotonin methyltransferase.

To study the development-specific variations in the expression pattern of genes encoding Ser and Mel biosynthetic enzymes, corresponding gene expression profiles were retrieved from Genevestigator (details given in Materials and Methods section). For some genes such as *AtT5H1*, *OsTDC3/6*, *OsASMT4*, *SbASMT2/15/18/26*, *SlTDC1/2/4* and *SlT5H3/4/5*, and *ASMT* genes from tomato (except *SlASMT9*), the data were not available. Differential expression was observed for most of the genes across various stages of plant development (Figure 4A,B). Notably, *AtASMT17* from Arabidopsis and *OsT5H1* from rice showed moderate to high levels of expression at almost all developmental stages in comparison to the other genes of that species (Figure 4A,B). However, in sorghum and tomato, *SNAT* genes, namely *SbSNAT1* and *SlSNAT2*, were found to be most highly expressed.

Interestingly, transcript levels of *AtASMT13* and *AtASMT14* were elevated during senescence, whereas those of *AtASMT17* decreased in the senescing phase (Figure 4A). On the other hand, *AtASMT9* was expressed at relatively higher levels during the early germination and vegetative phases, with its expression decreasing thereafter. Likewise, *AtSNAT1* expression was higher during the flowering and silique formation stage but decreased during maturation and senescence. The *AtTDC* genes accumulated to relatively lower levels with little variations being detected across different developmental stages. Furthermore, expression of certain genes from tomato was stage-specific, although they were expressed at relatively lower levels in comparison to the corresponding genes from Arabidopsis (Figure 4A). On the other hand, expression of *SlTDC5*, *SlT5H6* and *SlSNAT2* was highly induced during fruit ripening, while *SlT5H1* was induced during flowering and fruit formation. Similarly, *SlSNAT2* transcript levels were found to be specifically higher in shoot tissues and during the flowering stage (Figure 4A). 

In rice, greater variations in Ser and Mel gene expression was observed in various developmental stages when compared to the dicots (Figure 4B). For example, expression of *OsSNAT1* and *OsASMT8/9* was significantly induced at the heading stage, while the expression of *OsTDC4* and *OsT5H1* was higher during the dough stage, and *OsTDC5* showed specifically reduced expression during the milk stage (Figure 4B). In sorghum, gene expression was examined at the seedling, stem elongation, booting, flowering and dough stages. *SbTDCs*, *SbT5H1* and *SbSNAT1* genes showed almost no developmental variations, with the expression levels of *SbSNAT1* being the highest throughout (Figure 4B). While *SbASMT16*, *SbASMT20*, *SbASMT22* and *SbASMT24* were particularly induced in the seedling and stem elongation stages, *SbASMT20* and *SbASMT22* were found to be significantly upregulated in the seedling, stem elongation, flowering and dough stages (Figure 4B). In comparison to sorghum, rice displayed more prominent expression of genes encoding Ser and Mel biosynthetic enzymes during different developmental stages (Figure 4B).

Furthermore, to understand the regulation of genes encoding enzymes for Ser and Mel biosynthesis in different tissues of selected model plants, the corresponding expression profiles were analysed. In the case of sorghum, expression data was available for only root and shoot tissues. Among the *TDC* transcripts analysed in the study, *OsTDC5* had the highest expression, its transcript levels being higher in all the tissues analysed (Figure 4C), whereas *OsTDC1* was highly expressed in shoot tissues and seedlings. Other *TDC* transcripts exhibited moderate expression levels with little variation among different tissues (Figure 4C). On the other hand, *OsTDC4* and *AtTDC1* were specifically expressed in root and shoot tissues, respectively (Figure 4C). Among the *T5H* transcripts, *OsT5H1* expression was significantly higher across all tissues, whereas the *T5H* transcripts in tomato showed relatively lower expression levels than those in other species. Moreover, we observed that the expression levels of *SNAT* genes were generally higher in shoot tissues and seedlings compared to root tissues and inflorescence, except for *SNAT* genes from tomato (Figure 4C). Root- or shoot- specific variation was observed for most of the *ASMT* transcripts across all species (Figure 4C). In the case of rice, *OsASMT11* was highly expressed in root, whereas *OsASMT8/9/15/19* were induced more in shoot and seedlings (Figure 4C). Likewise, *SbASMT10, OsASMT12/14/24* and *OsASMT28* were highly induced in root whereas *SbASMT20* was distinctly upregulated in shoot (Figure 4C). Notably, in Arabidopsis, *AtASMT4* was expressed at higher levels in shoot and seedlings, whereas in root, *AtASMT1/9/13* and *AtASMT14* showed elevated expression (Figure 4C). Interestingly, *AtASMT9* and *AtASMT17* showed significantly higher expression in all the tissues analysed, while *AtASMT7* and *AtASMT8* appeared to be flowering-specific as they were relatively more expressed in inflorescence tissues (Figure 4C). Overall, the transcript levels of the genes encoding Ser and Mel biosynthetic enzymes were differentially regulated during development and in various tissues in the four plant species. It is likely that the different isoforms function during different developmental stages or in different tissues to exert a precise control over Ser/Mel biosynthesis during plant growth. 

### 2.5. Genes Involved in Ser and Mel Biosynthesis Have Rhythmic Expression in Response to Light/Dark Conditions

**Figure 5:**
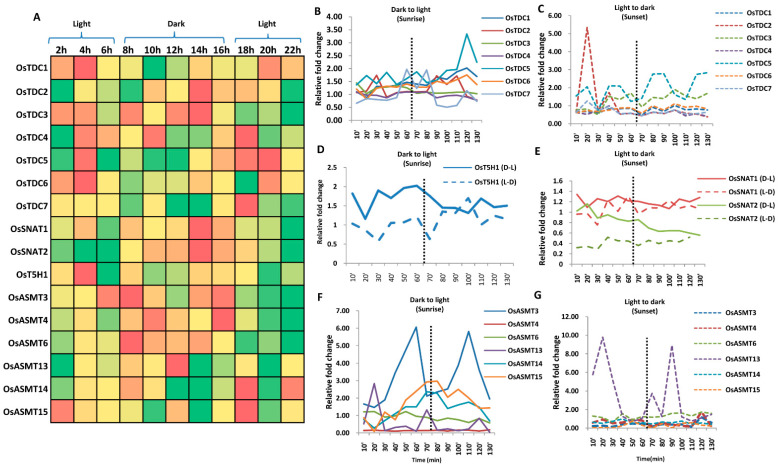
Regulation of genes encoding Ser and Mel biosynthetic enzymes under light and dark conditions in rice. (**A**) Diurnal regulation of genes encoding Ser and Mel biosynthetic enzymes. Line diagram depicting expression profile of Ser and Mel biosynthetic genes as (**B**,**C**) *OsTDCs*, (**D**) *OsT5H*, (**E**) *OsSNATs* and (**F**,**G**) *OsASMTs,* during sunrise and sunset. D→L represents transition from dark to light and L→D represents transition from light to dark. The dotted line represents the transition phase during sunrise and sunset. Transcript data for genes encoding Ser and Mel biosynthetic enzymes was obtained from the RiceXPro database. Red and green colour indicates higher and lower expression values. Yellow colour indicates intermediate values. Temporal fold change in expression was calculated relative to the 0 min/hr time point (i.e., starting time point) for all data shown here.

The levels of Ser and Mel are known to be affected by light [25,38], hence, the transcript levels of rice genes encoding Ser and Mel biosynthetic enzymes were assessed for 24 h in order to monitor fluctuations in their expression over the day–night cycle, using data from public repositories. A distinct expression pattern for most of the genes was witnessed during the initial stages of plant development. *OsTDC1* was induced in the light phase, whereas *OsTDC2* was found to be up-regulated under dark conditions (Figure 5A). Furthermore, expression of *OsTDC3* and *OsTDC4* appeared to be independent of light and dark conditions but fluctuated over a 24 h period, while *OsTDC6* and *OsTDC7* transcripts were found to be somewhat light-responsive (Figure 5A). As in *OsTDC3/4*, expression of *OsT5H1* was also not strictly dependent on light/dark conditions (Figure 5A). Among the genes encoding Mel biosynthetic enzymes, *OsSNAT1* and *OsSNAT2* were typically induced in the dark. Similar expression patterns were noted for *OsASMT4*/*6* and *OsASMT13*, with the exception of *OsASMT15*, which was light-inducible (Figure 5A). These expression patterns (Figure 5A) correlate with Ser production, which is mediated during the light phase, whereas Mel production is favoured under dark conditions [25,38].

Because genes encoding Ser and Mel biosynthetic enzymes showed alterations during a 24 h period or by light/dark regimes, we subsequently examined their expression levels during transition from dark to light conditions, and vice versa (i.e., sunrise and sunset). *OsTDC5* exhibited significant up-regulation during sunrise, whereas all other *OsTDC*s maintained basal expression levels or showed marginal fluctuations (Figure 5B). Furthermore, *OsTDC2* transcripts were significantly upregulated during early sunset (Figure 5C). Overall, rhythmicity in expression (over 10 min intervals) was more prominent for *OsTDC5* during both phases (Figure 5C). *OsT5H1* and *OsSNAT1* transcript levels were found to be marginally altered, whereas *OsSNAT2* exhibited a gradual decline in expression level, after sunrise (Figure 5D,E). No distinct pattern in *OsSNAT2* expression was observed during sunset (Figure 5E). In the case of *OsASMTs*, a rhythm in expression could be observed for all genes during sunrise (Figure 5F). Among all the genes, *OsASMT3* was highly upregulated, exhibiting a rhythmic pattern of up- and down-regulation (Figure 5F). However, *OsASMT15* expression levels were gradually induced during sunrise (Figure 5F). Furthermore, *OsASMT13* showed greatest fluctuations in expression levels during sunset (Figure 5G). The above data implies that the expression levels of genes encoding enzymes for Ser and Mel biosynthesis are altered by light/dark conditions, exhibiting rhythmic pattern. We believe that some transcripts might be playing crucial roles during the light phase, whereas others orchestrate gene regulation sequentially during darkness. In fact, this light/dark-mediated regulation of Ser and Mel biosynthesis may offer a mode by which various developmental processes otherwise dependent on light/dark conditions (or indirectly by temperature) such as flowering, vernalisation, dormancy and even stress, are controlled by Ser and Mel. 

### 2.6. Genes Encoding Enzymes for Ser and Mel Biosynthesis in Plants Are Highly Stress-Responsive

**Figure 6:**
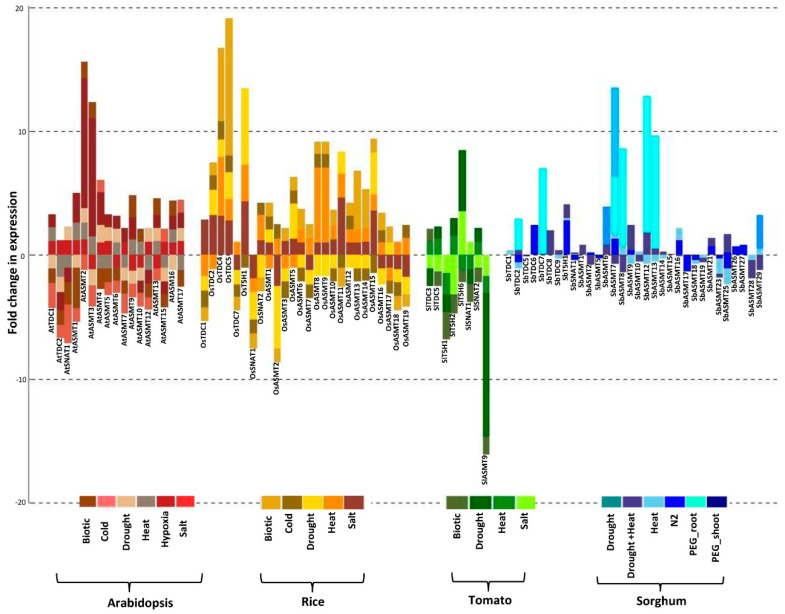
Stress-mediated regulation of genes encoding Ser and Mel biosynthetic enzymes in different plant species. Stacked bar plots depicting expression profiles of genes encoding Ser and Mel biosynthetic enzymes in rice, Arabidopsis and tomato were obtained from the publicly available Genevestigator database. Perturbation data for the Sorghum genes was obtained from the Expression Atlas database. Stacked bar plots were generated using R package. Levels of expression have been indicated by the Y axis. The colour bars at the bottom of the graph represent the different stress conditions.

For comprehensively investigating the stress-mediated perturbations in genes encoding enzymes for Ser and Mel biosynthesis, their expression levels were analysed in all four plant species using publicly available stress-related datasets from Genevestigator. Genes encoding Ser and Mel biosynthetic enzymes were indeed found to be stress-regulated in all four plant species. In response to salinity, most of the genes encoding Ser and Mel biosynthetic enzymes were induced in rice and Arabidopsis, but in tomato, salinity had an overall repressing effect on gene expression (Figure 6). Likewise, drought conditions also had a somewhat similar effect on the expression of Ser and Mel pathway genes in different species. In Arabidopsis, most of the *AtASMTs* were drought-inducible, with *AtASMT2* and *AtASMT3* exhibiting a 10.5- and 9.5-fold change, respectively, being highest among all genes. The rice *OsASMT*s exhibited a differential response to drought, with the majority of genes being downregulated. Tomato and sorghum genes also exhibited perturbations in response to drought, with some showing induction, while others exhibited down regulation in expression (Figure 6). 

In response to heat, most of the Arabidopsis and rice genes encoding Ser and Mel biosynthetic enzymes showed a mixed response, with at least one gene of each family showing heat-inducibility. The *AtTDC* and *OsT5H* genes emerged as exceptions because all genes of the corresponding families were heat-downregulated (Figure 6). Similar observations were made in tomato, as *SlT5H6* and *SlASMT9* exhibited heat-mediated repression. Interestingly, in sorghum, heat stress led to a manifold increase in the expression of several genes such as *SbTDC2*, *SbTDC7*, *SbASMT7/8/12* and *SbASMT13.* Heat stress in combination with drought stress led to up-regulation of *SbASMT6* and *SbASMT29* (Figure 6). 

PEG treatment given to impose osmotic stress led to down-regulation of most of the *SbASMT* genes in sorghum but induced the expression of most of the *SbTDCs* and *SbT5H1* in both root and shoot tissues. In Arabidopsis, low temperature majorly repressed the expression of genes encoding Ser and Mel biosynthetic enzymes, whereas in rice, it induced the expression of most of the genes of the Ser and Mel pathway. Further, *SbASMT7* was found to be induced under nitrogen starvation conditions and was the only gene to be affected by such conditions in sorghum.

Interestingly, genes encoding Ser and Mel biosynthetic enzymes were also affected by hypoxia, as observed in Arabidopsis, with most of the genes being induced under hypoxic conditions, but some genes, such as *AtTDC2, AtSNAT1* and some *AtASMTs*, were downregulated (Figure 6). Besides abiotic stress, the effect of biotic stress factors on gene expression was also determined. We found that biotic factors largely downregulated the expression of genes in Arabidopsis. In contrast, expression of most of the rice Ser and Mel biosynthetic genes was enhanced under biotic stress. In tomato, except for *SlT5H6* and *SlSNAT1*, all other genes were downregulated in response to biotic stress (Figure 6). Overall, genes encoding enzymes for Ser and Mel biosynthesis were found to be diversely regulated under different stress conditions. Importantly, the stress-mediated expression profile of these genes was found to be dependent on the plant genotype, and no uniformity in expression patterns in response to any particular stress could be observed across different plant species. 

### 2.7. Phytohormones Regulate the Expression of Genes Encoding Ser and Mel Biosynthetic Enzymes in Plants

**Figure 7:**
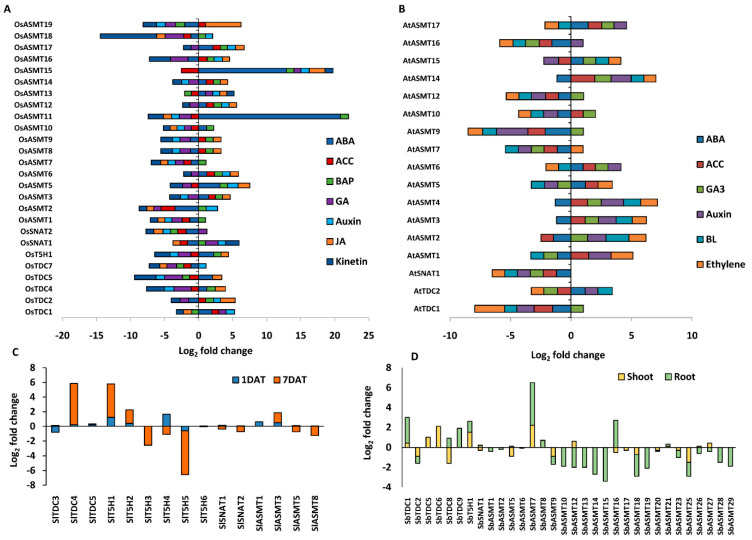
Phytohormone-mediated regulation of genes encoding Ser and Mel biosynthetic enzymes in different plant species. Stacked bar plots depicting expression profile of genes encoding Ser and Mel biosynthetic enzymes in (**A**) rice, (**B**) Arabidopsis and (**C**) tomato (Auxin-treated) was obtained from the publicly available Genevestigator database and supplementary data of corresponding studies. Perturbation data for the genes in (**D**) Sorghum (ABA-treated) was obtained from the Expression Atlas database. DAT, days after auxin treatment. ABA, Abscisic acid; ACC, 1-aminocyclopropane-1-carboxylic acid; BAP, Benzylaminopurine; GA, Gibberellic acid; JA, Jasmonic acid; IAA, Indole acetic acid; BL, Brassinolide.

Ser and Mel are in direct and continuous crosstalk with several plant hormones, and their role in modulating the endogenous phytohormone levels is also known [18,39]. Hence, the effect of phytohormones in regulating the expression of genes encoding Ser and Mel biosynthetic enzymes in different plant species has been explored. For this, the expression profile of genes was retrieved from publicly available datasets (Figure 7A–D). In rice, we noticed that jasmonic acid (JA) and benzylaminopurine (BAP) induced the expression of most of the genes encoding Ser and Mel biosynthetic enzymes. In fact, the expression of *OsASMT1* and *OsASMT7* was induced solely by BAP. 

ABA also had an inductive effect on the expression of most of the rice genes encoding Ser and Mel biosynthetic enzymes. *OsASMT11* (20-fold), *OsASMT15* (12.8-fold) and *OsASMT5* (3-fold) were found to be among the maximally induced genes in response to ABA (Figure 7A). However, ABA repressed the expression of majority of the genes in Arabidopsis, except *AtTDC2*, *AtASMT5*, *AtASMT6*, *AtASMT15* and *AtASMT17* (Figure 7B) but up-regulated the expression of genes encoding Ser biosynthetic enzymes in sorghum. Notably, *SbASMT7* and *SbASMT16* were the only genes belonging to the Mel biosynthetic pathway which were induced by ABA (Figure 7D). Induction of genes encoding Ser and Mel biosynthetic enzymes was higher in root tissues than in shoot tissues under ABA treatment (Figure 7D). 

Gibberellic acid (GA) also had contrasting effects on the expression of the Ser and Mel biosynthetic genes in rice and Arabidopsis. Notably in rice, GA repressed the expression of most of the biosynthetic genes, while the opposite trend was observed in Arabidopsis, as GA induced the expression of *AtTDC1* and most of the *AtASMTs* (Figure 7B). On the other hand, auxin did not significantly affect the expression of genes encoding Ser and Mel biosynthetic enzymes in both rice and Arabidopsis (Figure 7A,B). However, in tomato, prolonged exposure to auxin repressed the expression of *SlASMTs* (Figure 7C). Other hormones, such as kinetin, brassinolide and ethylene, mostly reduced the expression of genes encoding Ser and Mel biosynthetic enzymes. Thus, it is evident from the analysis that genes for Ser and Mel biosynthesis are indeed regulated by one or more different types of plant hormones across specific plant species. While some phytohormones were found to significantly alter the expression levels of these genes, others caused minimal perturbations, reinstating the fact that response to hormones was also plant specific.

### 2.8. Genes Involved in Ser and Mel Biosynthesis in Rice Are Affected by Light Availability and Stress as Determined by qRT-PCR

**Figure 8:**
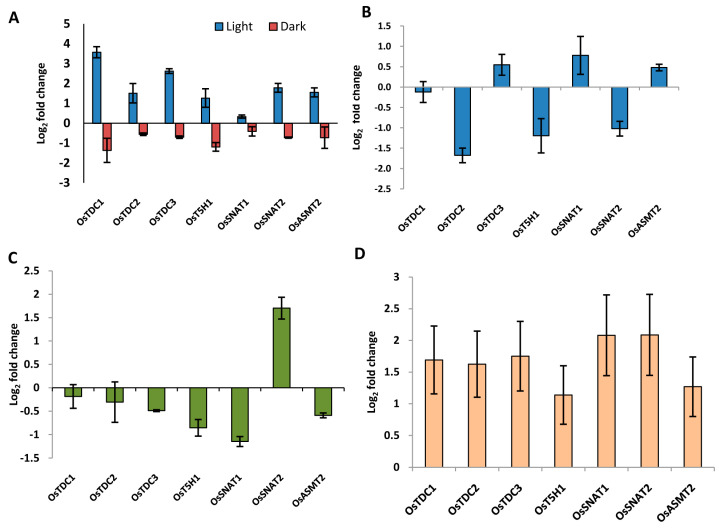
Expression analysis of genes encoding Ser and Mel biosynthetic enzymes in rice in response to light/dark and abiotic stress treatments by qRT-PCR. Expression profiling of genes encoding Ser and Mel biosynthetic enzymes in 8-day-old rice seedlings exposed to (**A**) light (100 µmol/m^2^/s)/dark conditions, (**B**) salinity (200 mM NaCl), (**C**) heat (42 °C) for 6h and (**D**) drought (water withheld) for 24 h. Logarithmic changes in expression levels have been calculated with respect to the untreated control. Normalization was carried out with respect to the housekeeping gene, *elongation factor 1 alpha* (*eEf-1α*).

To validate the influence of light availability and stress on the expression of genes encoding Ser and Mel biosynthetic enzymes, we next determined the relative transcript abundance of a few rice genes by qRT-PCR, in seedlings subjected to 6h of light/dark conditions. The genes were selected based on their confirmed role in Ser and Mel biosynthesis as deduced from the published literature. The *TDC* genes were found to be highly upregulated under light (Figure 8A). Among all genes, *OsTDC1* showed maximum induction (3.6-fold) during the light phase, followed by *OsTDC3* (Figure 8A). However, all genes were downregulated in darkness (Figure 8A).

To assess the stress inducibility of these genes, eight-day-old rice seedlings were subjected to abiotic stress conditions by imposing salinity, drought and heat treatments for 6 h. The transcripts of *OsTDC2*, *OsT5H1* and *OsSNAT2* were found to be downregulated (more than 1.5-fold) under salinity stress (Figure 8B). Notably, under heat stress, all the genes showed down-regulation in their expression, except for *OsSNAT2*, which was 1.7-fold upregulated (Figure 8C). Interestingly, all the analysed genes were also highly induced under drought stress treatment (Figure 8D). These results are indicative of stress-responsiveness of the Ser and Mel biosynthetic pathway in rice. Importantly, we could observe a correlation with publicly available expression data for many of the genes. The lack of correlation between the expression profiles of the others could possibly be due to differences in experimental conditions as these genes also exhibit rhythmicity in expression. These qRT-PCR experiments, therefore, revealed that rice genes encoding Ser and Mel biosynthetic enzymes are indeed regulated by different stress conditions and also exhibit modulation under particular light/dark regimes.

### 2.9. miRNAs Regulate Expression of Genes Involved in Ser and Mel Biosynthesis

To understand whether the genes encoding Ser and Mel biosynthetic enzymes were also regulated by miRNAs, a computational prediction was carried out. This analysis identified a total of 40, 51, 57 and 7 putative miRNAs, which can target genes encoding Ser and Mel biosynthetic enzymes in Arabidopsis, rice, sorghum and tomato, respectively (Supplementary Table S3). One of the microRNAs, miR156 was conserved in all but Arabidopsis and the targeted *ASMT* gene family (Supplementary Table S4). However, other miRNAs such as miR159, miR393a, miR398a, miR447 and miR5635 specifically targeted the Arabidopsis genes but not the homologues in rice, sorghum and tomato. 

**Figure 9:**
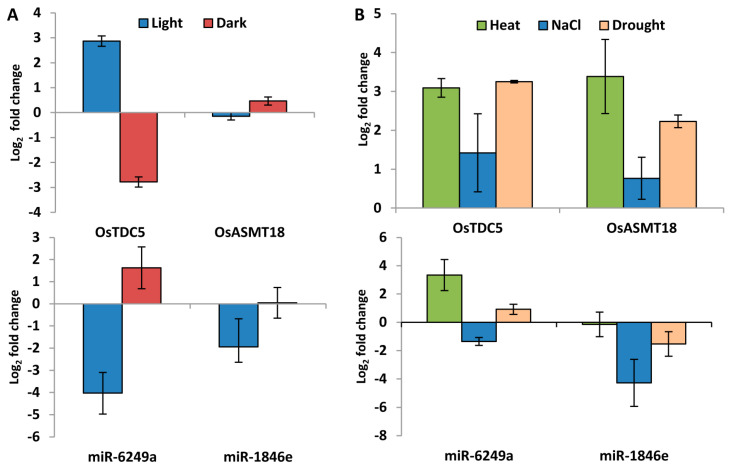
Light/dark and stress-mediated regulation of microRNAs and its corresponding target genes. Expression profiling of *OsTDC5* and *OsASMT18* and its corresponding miRNA under (**A**) light (100 µmol/m^2^/s)/dark conditions and (**B**) stress such as exposure to heat (42 °C), salinity (200 mM NaCl) for 6h and drought condition (water withheld) for 24 h.

Further, using a highly stringent criteria for prediction, the miRNA target pairs (expectancy value *e* < 3), *osa-miR6249a-OsTDC5* and *osa-miR1846e-OsASMT18*, were finally selected for qRT-PCR validation. Expression profiling of these miRNA-target pairs under a light/dark regime in rice seedlings revealed exactly opposite expression patterns (Figure 9A). *OsTDC5* was up-regulated in light and down-regulated in dark, while *osa-miR6249a* was down-regulated in light and up-regulated in dark (Figure 9A). However, *OsASMT18* expression was not significantly altered under light/dark conditions, but *osa-miR1846e* expression was significantly down-regulated during light (Figure 9A). Furthermore, analysis of the expression profiles of miRNA-target pairs under heat, salinity and drought stress in rice seedlings revealed significant up-regulation of *OsTDC5* and *OsASMT18* in almost all the stress conditions analysed (Figure 9B). While *osa-miR6249a* expression was up-regulated under heat and down-regulated under salinity stress, *osa-miR1846e* expression levels were significantly down-regulated under salinity and drought stress conditions (Figure 9B). Expression of *miR6249a-OsTDC5* pairs exhibited opposite regulation, specifically under salinity and *osa-miR1846e-OsASMT18* under salinity and drought. However, both miRNAs did not seem to regulate the heat stress response of the respective genes. The above results also suggested that selected miRNA-target pairs are differentially regulated under specific light/dark regimes and different stress conditions. This evidence implies a fine-tuned regulation of miRNAs and their target gene (encoding Ser and Mel biosynthetic enzymes) pairs in mediating light and stress-related responses in rice.

## 3. Discussion

In order to compensate for the lack of mobility, plants have evolved an intricate network of signals which sense and respond to the surrounding environment. Intriguingly, despite being devoid of a central nervous system, plants are still capable of producing certain signalling molecules similar to those found in the mammalian nervous system, referred to as neurotransmitters. Ser (or 5-hydroxy-tryptamine) and Mel (or N-Acetyl-5-methoxytryptamine) are examples of the same. These have been recognized as novel plant growth regulators possessing a plethora of physiological functions in plants, ranging from development to abiotic stress responses [28,40]. These molecules are in direct crosstalk with several plant hormones and interrelated with crucial signalling pathways [18,40]. Considering the important, diverse and multifaceted roles of these signalling molecules in plant life, it is worth identifying genes encoding for enzymes mediating Ser and Mel biosynthetic pathway in plants. The major biosynthetic pathway for these indoleamines includes four enzymes, namely, tryptophan decarboxylase (*TDC*), tryptamine 5-hydroxylase (*T5H*), serotonin N-acetyl transferase (*SNAT*) and acetyl-serotonin methyltransferase (*ASMT*). Of them, only selected gene families have been functionally characterised to date, such as *TDC*s in tomato, citrus and rice, and *ASMT*s in tomato and pepper [8,10,11,12,41]. Therefore, in the present study, we have carried out a comprehensive genome-wide analysis to identify possible gene family members of all the four enzymes of the Ser and Mel biosynthetic pathway in rice, Arabidopsis, tomato and sorghum. Of these, the *ASMT* gene family comprises maximum members, whereas *T5H* is encoded by a single gene across plant species, except in tomato, which has six putative members. Our phylogenetic analysis revealed each enzyme to be clustered into different groups, consisting of proteins from either the same or different species, indicating diversification of the proteins.

The presence of Ser and Mel is ubiquitous [3] and is also conserved across plants [42]. Chloroplasts and mitochondria are considered to be the original sites for the synthesis of Ser and Mel in plants, inheriting Ser and Mel biosynthetic abilities from their bacterial ancestor [43]. In particular, the presence of genes encoding Ser and Mel biosynthetic enzymes in alpha-proteobacteria and cyanobacteria is important, as they are considered to be the ancestors of the plant organelles. In correlation with this fact, *SNAT* proteins from rice, one of the key enzymes in Mel biosynthesis, shows substantial homology at the gene level to that of the cyanobacterium *Synechocystis* and, likewise, is plastidially located [44]. In fact, all *SNAT* proteins characterized to date are strictly chloroplastic, reaffirming their cyanobacterial heritage. However, our analysis has suggested that different genes of the Ser and Mel biosynthetic pathway may have variable localization. While *TDC* genes majorly localise in the cytoplasm (except for Arabidopsis *TDCs*, which were mostly chloroplastic), both *T5H* and *SNAT* genes were predicted in the chloroplast in all four plant species, in agreement with the endosymbiosis theory. ASMTs, however, exhibited highly variable sub-cellular localization patterns within the four plant species. While members of the *AtASMT* and *SbASMT* gene family were predicted to be cytoplasmic, only a very few members of the *OsASMT* and *SlASMT* gene family showed similar localization patterns. Some members of the rice and tomato *ASMT* gene family were indeed found to localize in the cytoskeleton and nucleus. Preliminary work in rice and other recent reports also show localization of the biosynthetic enzymes in cytoplasm, chloroplast and mitochondria [9,44,45]. 

Currently, limited information is available regarding the tissue-specificity of Ser. However, there is some evidence to suggest that its levels are highest in the aerial parts of plants, such as in potato and *Ligisticum spp* [46,47]. In addition, Ramakrishna et al. [48] found Ser to be localised in the mature roots and stems of *Coffea canephora* Pierre ex. Using quantum dots, Mel and Ser uptake in the axenic roots of St. John’s wort has also been studied. Under optimal conditions, Mel is absorbed through epidermal cells, travels laterally across the root cortex and accumulates in endodermal tissues and rapidly dividing pericycle cells, while Ser is absorbed by cells proximal to the crown and is rapidly transported towards the root tip [49]. Our analysis of the transcript abundance of putative *TDC* and *T5H* genes involved in Ser biosynthesis reveals lesser tissue-specific variations across the four different plant species. In fact, *OsT5H1* and *OsTDC5* are constitutively expressed in all the tissues. The other *OsTDC* genes, along with *AtTDC*, *AtT5H*, *SbTDC*, *SlTDC* and *SlT5H* genes show moderate to high level of expression in the different types of tissues. Likewise, the *SNAT* and *ASMT* genes involved in Mel biosynthesis are also expressed in all tissue types in rice, Arabidopsis, sorghum and tomato. While the expression levels of some genes vary across tissues, others, such as *OsSNAT1*, *SbSNAT1* and *AtASMT17*, showed constitutive expression, suggesting Mel to be distributed throughout the plant organs, an observation which is supported by studies on various edible and medicinal plants [50,51,52].

Ser and Mel probably exist in a balance, similar to that established for auxin and cytokinin, due to their close biosynthetic relationship. While Mel is suggested to behave in a similar manner to auxin, Ser acts in the same way as cytokinin, implicating its role in organogenesis, morphogenesis and the growth and development of plants [6]. Furthermore, transcript levels of the majority of genes encoding Ser and Mel biosynthetic enzymes are altered at specific developmental stages. For instance, *AtTDC1* is upregulated at the seed germination stage, *SlT5H6* at the fruit ripening stage and *OsTDC1* at the heading stage, whereas *SbASMT14* is specifically induced at the booting stage of development. However, some genes, such as *AtASMT7* and *OsT5H1*, are induced at all stages of plant development. Besides flowering and morphogenesis, the role of Ser is also well established in senescence [53]. In fact, Kang et al. [54] reported enhanced accumulation of Ser in rice leaves undergoing senescence, its synthesis being closely coupled with the transcriptional induction of its biosynthetic genes, especially *TDC*. In agreement with this, we found both *AtTDC1* and *AtTDC2* to be induced under senescence, with expression levels of *AtTDC1* being higher in comparison to *AtTDC2*. Notably, *ASMT* genes, especially *AtASMT13* and *AtASMT14*, also showed enhanced expression under senescence. This is particularly interesting because only very few studies have shown the regulation of Mel biosynthesis in promoting senescence [14,55]. Most studies have focussed on the exogenous application of Mel in delaying aging [56,57,58]. The senescence-specific induction of genes encoding Ser and Mel biosynthetic enzymes, therefore, suggests the specific role of Ser and Mel in senescence.

Furthermore, Ser biosynthesis is differentially induced in response to different wavelengths of light [59]. Exposure to red light leads to immediate accumulation of Ser in *Scutellaria,* which decreases over 7 days. In contrast, Mel is detected in plants grown under blue light [60]. Using a short-day flowering plant, *Chenopodium rubrum*, Kolářet al. [61] showed that light suppresses Mel biosynthesis in plants. However, Murch et al. [62] reported that light significantly stimulates Mel production in St. John’s wort. Our assessment of rice Ser and Mel encoding genes through qRT-PCR are in agreement with the results of Murch et al. [62]. In our analysis, we found all members of the *OsTDC*, *OsT5H*, *OsSNAT* and *OsASMT* gene families to be up-regulated in response to 6h light exposure subsequent to prolonged (for 2 d) darkness. On the contrary, data retrieved from public repositories indicate *OsSNAT* and *OsASMT* expression to be more pronounced in darkness. We believe that a change of growth cycle in our experiment has led to this difference in regulation of Ser and Mel biosynthetic genes as short/long day light cycles also affect Mel accumulation. In fact, the endogenous levels of Mel have been shown to vary in a typical diurnal pattern in Arabidopsis [28]. 

**Figure 10:**
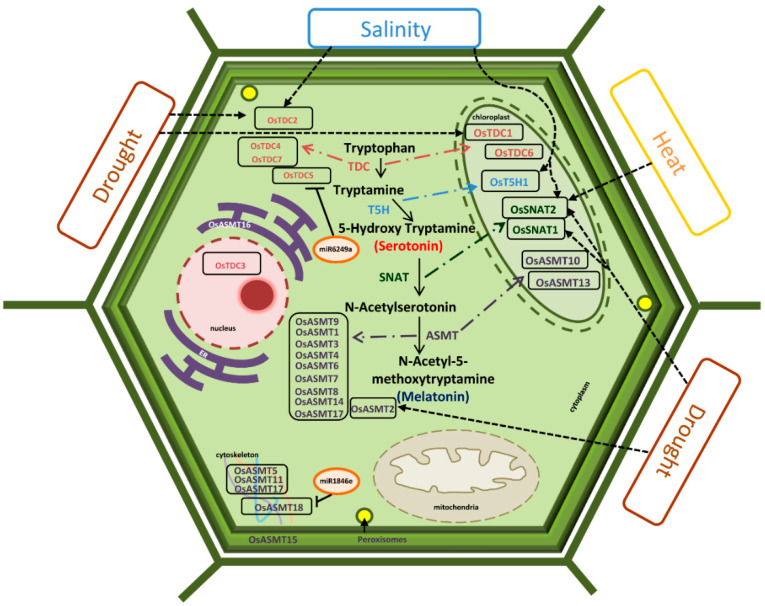
Model summarizing the members of Ser and Mel biosynthetic pathway of rice, highlighting their regulation. The crosstalk between genes encoding enzymes for Ser and Mel biosynthesis (identified by genome-wide analysis) harboured in different sub-cellular compartments, have been illustrated under stress conditions, based on transcript profile validations. The miRNA-based regulation of specific gene family members has also been indicated. The black dotted arrows depict the stress-inducibility of specific transcripts regulating Ser and Mel biosynthesis under various stress conditions, as validated by qRT-PCR. Solid black lines depict regulation of transcripts with specific miRNAs. Coloured arrows depict the genes encoding the enzymes involved in the Ser and Mel biosynthesis.

Furthermore, Ser and Mel also play an important role in imparting stress tolerance to plants (Figure 10). Ser and Mel mediate abiotic stress tolerance via their antioxidative defensive role, which leads to the detoxification of ROS and reactive nitrogen species (RNS) [63,64,65]. There is a wealth of information related to the effects of exogenous application of Ser and Mel in mitigating abiotic stresses in plants [32,66,67,68,69]. We also found genes encoding Ser and Mel biosynthetic enzymes to be maximally induced in response to drought conditions as well as upon ABA treatment, thereby reinforcing the role of ABA in the regulation of Ser and Mel levels under drought conditions. In addition, the available literature also highlights the role of these metabolites in biotic stresses such as herbivory and pathogen attack [70,71,72]. Mel induces SA and NO biosynthesis, acting upstream of the defence signalling pathway, thereby stimulating disease resistance because of co-action [73]. In agreement with this, we found most of the genes encoding enzymes for Ser and Mel biosynthesis in rice to be upregulated in response to both biotic stress and JA.

Importantly, the response of Ser and Mel biosynthetic genes to hormone treatments was found to be highly variable across four species. While ABA induced gene expression in monocots, the same had a repressive effect in Arabidopsis. However, GA showed a reverse response in both species, where it repressed gene expression in rice but induced it in Arabidopsis. Previous studies report that Mel alters both these hormone levels [18]. It up-regulates ABA catabolism in cucumber seedlings (under salinity) and apple leaves (under drought), whereas it increases ABA content in the monocot barley (under drought/cold stress) [74,75,76]. On the other hand, GA biosynthesis is enhanced in cucumber seedlings in response to Mel [74,75]. Thus, an intricate feedback regulatory mechanism exists in plants to control Ser and Mel levels. 

Interestingly, miRNAs are also involved in the regulation of Ser and Mel gene expression in plants. Mel is known to regulate miRNA gene expression in plants under certain conditions [77], but no report of miRNA–mediated regulation of Ser and Mel gene expression is yet available. Nevertheless, we could predict 51 unique miRNAs regulating Ser and Mel biosynthetic genes across the four plant species, of which, miR156 was found to be conserved in all species except Arabidopsis. However, some miRNAs, such as miR393 and miR398, could be identified in only Arabidopsis, suggesting that, possibly, miRNA regulation of these genes either originated relatively recently or was gradually lost in some of the plant species. The miR398 is also known to mediate oxidative and heavy-metal stress tolerance in plants [34,35], whereas miR393 has been shown to target the ABF2/TIR transcription factor, which in turn regulates auxin biosynthesis [78]. Furthermore, we found *OsTDC5* and *OsASMT18* to be regulated by miR6249a and miR1846e, respectively, under light/dark conditions and even under salinity and drought stress. Interestingly, both these miRNAs have been reported to be drought-responsive [79]; *miROs1846e* was indeed found to positively regulate *OsASMT18* under drought conditions.

Therefore, through this study, we have been able to gain crucial insights into the Ser and Mel biosynthesis pathway in plants. Indeed, expression patterns of these biosynthetic genes is regulated by stress conditions in plants and even exhibits tissue and developmental variations, in corroboration with diversity in their actions. Furthermore, similar to animals, these genes also exhibit light/dark cycle-mediated alterations in plants. Collectively, Figure 10 summarizes the information regarding rice genes encoding Ser and Mel biosynthetic enzymes and their regulation. Our study has, thus, generated a knowledge repository and paved the way for future investigations, which will enable us to gain deeper insights into the diverse regulatory roles of genes encoding enzymes for Ser and Mel biosynthesis in selected model plants.

## 4. Materials and Methods

### 4.1. Identification of Ser and Mel Biosynthetic Pathway Genes and Proteins in Selected Model Plants

Protein sequences for enzymes mediating Ser and Mel biosynthesis were obtained by domain search. The pyridoxal decarboxylase domain (PF00282) was used to search for the TDCs and p450 domain (PF00067) for *T5H*, whereas the sequences of the putative proteins involved in biosynthesis of Mel were retrieved by searching the acetyltransferase domain (PF00583) for SNATs and the dimerization domain (PF08100) and the methyltransferase domain (PF00891) for ASMTs, via Phytozome12 (https://phytozome.jgi.doe.gov/pz/portal.html; accessed on 3 February 2019). The obtained sequences were filtered based on alignment with previously characterized proteins of the Ser and Mel biosynthetic pathway (namely, *OsTDC1/2/3*, *OsT5H*, *OsSNAT1/2* and *OsASMT1*/2/3) in rice [15] via the NCBI Blast2 program using default parameters. For monocots, proteins were selected at ≥70% query coverage and ≥50% percentage identity at *e*-val threshold ≤ e^−30^. For dicots, selection criteria were relaxed, with sequences having ≥50% query coverage and ≥40% percentage identity being considered. Exceptions to this were *At*ASMT proteins, where protein sequences having ≥30% percentage identity were selected because no hits were found at the ≥40% identity threshold. All the sequences have been provided in Supplementary File S1. Information on genomic coordinates, location and length of transcripts, CDS and protein lengths were obtained from the Phytozome database. The molecular weight and isoelectric point of putatively identified proteins were predicted via the ExPaSy online tool (http://web.expasy.org/protparam/; accessed on 15 March 2019).

### 4.2. Analysis of Protein Sequences, Localization and Phylogenetic Tree Construction

The domain architecture of all four types of proteins, viz., *TDC*, *T5H*, *SNAT* and *ASMT*, were retrieved via HMMeR (https://www.ebi.ac.uk/Tools/hmmer/search/hmmscan; accessed on 10 April 2019). Subcellular localization of proteins was predicted using WoLF PSORT (https://wolfpsort.hgc.jp/), Localizer (http://localizer.csiro.au/) as well as ChloroP (http://www.cbs.dtu.dk/services/ChloroP/) software (accessed on 2 September 2019).

The phylogenetic relationship of genes among different species was determined using Clustal Omega (https://www.ebi.ac.uk/Tools/msa/clustalo/; accessed on 7 May 2019). The phylogenetic trees were constructed using the neighbour-joining method and visualized in iTOL (https://itol.embl.de/upload.cgi; accessed on 16 July 2019).

### 4.3. Expression Profiling of Genes Encoding Ser and Mel Biosynthetic Enzymes Using Publicly Available Transcript Data

Expression of genes encoding enzymes mediating Ser and Mel biosynthesis was analysed in various anatomical structures and at different developmental stages in rice, Arabidopsis, sorghum and tomato, using publicly available expression data from Genevestigator (https://genevestigator.com/; accessed on 15 March 2019). For analysing diurnal/circadian variations in the expression profile of genes, the RiceXPro (https://ricexpro.dna.affrc.go.jp/publication.html; accessed on 15 June 2020) database was searched, and data were also retrieved from various experiments (GSE36043: Sunrise, GSE36044: Sunset, GSE39423: Diurnal cycle). The expression profile of genes was retrieved for rice leaf samples collected at 10 minute intervals from 3.50 am to 6 am for sunrise (D→L represents transition from dark to light) and from 5 pm to 8 pm for sunset (L→D transition). The temporal fold change in expression levels was calculated with respect to initial time points (3.50 am, 5 pm and 10:00 am for sunrise, sunset and diurnal variations, respectively). Abiotic/biotic stress and hormone-related expression profiles were retrieved via publicly available microarray data from Genevestigator (GSE15577, GSE37557, GSE5167, GSE19024, AtGenExpress dataset GSE6177, E-MEXP-2377, GSE26266, GSE29941, GSE8787 (Arabidopsis); GSE18361, GSE6901, GSE14275 (rice); GSE33177, GSE22304, GSE16401 (tomato)). The expression profile of tomato genes encoding Ser and Mel biosynthetic enzymes in response to phytohormone treatment was retrieved from supplementary material made available by Li et al. [80]. Normalized and curated stress-related RNA-sequencing data for genes encoding Ser and Mel biosynthetic enzymes of sorghum was obtained from the Expression Atlas repository (E-GEOD-30249 for ABA and PEG treatments, E-GEOD-54705 related to nitrogen tolerance conditions and GSE48205 for drought and heat stress data). Stacked bar plot and heatmaps were generated using R package (accessed on 11 October 2020) [81].

### 4.4. Computational Prediction of miRNAs Targeting Ser and Mel Genes in Plants

The identified genes encoding Ser and Mel biosynthetic enzymes in Arabidopsis, rice, tomato and sorghum were used as query sequences, and their corresponding miRNAs were searched in miRbase version 21 (accessed on 24 June 2019) for all the plant species under study using the previously reported pipeline [34,82]. For prediction of all the miRNA targets, psRNA Target tool was used (http://plantgrn.noble.org/psRNATarget/; accessed on 18 July 2019).

### 4.5. Plant Materials and Growth Conditions

IR64 rice (*Oryza sativa cv. indica*) seeds supplemented with Yoshida media were grown in sterile germination rolls under controlled conditions in the growth chamber for 4 days in light followed by two days in dark at 28 °C. On the 8th day, rice seedlings were subjected to light and dark conditions for 6h, and tissue was harvested and stored at −80 °C until further use.

For stress treatments, eight-day-old rice seedlings grown at 28 °C were kept at 42 °C (for heat stress) or supplemented with Yoshida media containing 200 mM NaCl (salinity stress) for 6h, whereas for drought stress, water was withheld for 24 h. Seedlings kept in Yoshida media only served as control. Following stress treatment, tissue was harvested and stored at −80 °C until further use.

### 4.6. RNA Isolation and Quantitative Real-Time PCR (qRT-PCR) Analysis 

For qRT-PCR, total RNA was extracted using TRI reagent (Sigma-Aldrich, St. Louis, MO, USA) according to the manufacturer’s protocol. First-strand cDNA synthesis was carried out using a RevertAid first-strand cDNA synthesis kit (Thermo Fischer Scientific, Waltham, MA, USA). The primer sequences used in this study have been provided in Supplementary Table S5. The qRT-PCR analysis was performed as described elsewhere [83]. For normalization of transcripts, *elongation factor 1-alpha* (*EF-1a*) was used. Expression levels were calculated relative to untreated conditions. For determining expression of miRNA, cDNA synthesis was carried out using an miR-specific stem-loop primer via Superscript reverse transcriptase III (Invitrogen), and data normalization was carried out using the 18S rRNA internal control gene.

## 5. Conclusions

Ser and Mel govern key physiological processes and stress adaptations in plants. Through this study in selected monocot and dicot plants, we have uncovered the presence of multiple genes for each of the four enzymes involved in the Ser and Mel biosynthetic pathway. The expression profiles of these genes are specifically altered during development, in response to specific light/dark regimes, various stresses and phytohormones. Importantly, we have shown that these genes can be regulated post-transcriptionally via miRNAs. Because the members of each gene family differ in their biochemical and molecular features, they can confer functional specificity to Ser and Mel biosynthesis and, in turn, selectively regulate diverse physiological processes. Overall, this study encompasses the investigations in the context of a broad range of factors which modulate Ser and Mel biosynthesis in plants. The knowledge repository presented in this study can open new avenues crucial to comprehending the complex mechanistic regulations of the Ser and Mel pathway in plants.

## Figures and Tables

**Table 1:** Details of the genes and proteins involved in Ser and Mel biosynthesis in various model monocot and dicot plant species.

Name	Rice		Arabidopsis		Tomato		Sorghum	
	Genes	Proteins	Genes	Proteins	Genes	Proteins	Genes	Proteins
TDC	7	7	2	5	5	5	9	9
T5H	1	1	1	1	6	6	1	1
SNAT	2	2	1	3	2	2	1	1
ASMT	19	19	17	19	11	11	28	29

## Data Availability

The datasets supporting the conclusions of this article are included within the article and its additional files. The sequence data was obtained from Phytozome v12 (https://phytozome.jgi.doe.gov) for *Sorghum bicolor* and *Solanum lycopersicum*. For rice and *Arabidopsis,* sequence data was retrieved from the RGAP (http://rice.plantbiology.msu.edu/) and TAIR (https://www.arabidopsis.org/) databases, respectively.

## Supplementary Materials

The following are available online at https://www.mdpi.com/article/10.3390/ijms222011034/s1. Table S1: List of putative genes encoding Ser/Mel biosynthetic enzymes in Arabidopsis, rice, to-mato and sorghum. Table S2: Information regarding domain organisation of Ser/Mel biosynthetic enzymes. Table S3: List of predicted miRNAs targeting genes encoding Ser/Mel biosynthetic en-zymes in Arabidopsis, rice, tomato and sorghum. Table S4: Schematic representation of predicted miRs targeting Ser/Mel biosynthetic genes showing homology in Arabidopsis, rice, sorghum and tomato. Y (‘yes’) and N (‘no’) denote the presence or absence of sequence homology, respectively. Table S5: List of primers used in the study. File S1. Amino acid sequence of proteins involved in Ser and Mel biosynthesis in rice, Arabidopsis, tomato and sorghum.
